# Questionnaire data on perfectionism, flourishing and negative emotion symptoms of Chinese adolescents

**DOI:** 10.1016/j.dib.2020.105379

**Published:** 2020-03-05

**Authors:** Wenjie Duan, Yuan Guan

**Affiliations:** a Social and Public Administration School, East China University of Science and Technology, 130 Meilong Road, Xuhui District, Shanghai, China; b Guangdong University of Finance, Guangzhou, China

**Keywords:** Paper-and-pencil questionnaire, Adolescents, Perfectionism, Flourishing, Negative emotion symptoms

## Abstract

The data include 468 Chinese adolescents’ responses of the following psychological scales: (1) 45-item Big Three Perfectionism Scale (BTPS), (2) Chinese version of Flourishing Scale, and (3) 21-item Depression Anxiety Stress Scale (DASS-21). The data also comprises gender and age of the respondents. The samples were collected via interviews from a middle school in Sichuan Province, China. The dataset was obtained with paper-and-pencil questionnaire from May 2017 to October 2017. The data includes 368 males and 100 females (mean age 14.49, *SD* = 1.11; range from 13 to 17). The dataset can be used for cross-cultural analyses or exploratory studies.

**Table:** **Specification Table**

Subject	Social Work, Psychology, Social Sciences
Specific subject area	Individual differences, wellbeing, perfectionism, children and adolescents
Type of data	Tables
How data were acquired	Paper-and-pencil questionnaires
Data format	Raw, filtered, and summarized
Parameters for data collection	Chinese Adolescents (n =468) completed a battery of scales
Description of data collection	Cross-sectional
Data source location	Sichuan Province, Mainland China
Data accessibility	With the article
Related research article	Duan, W., He, C., Huang, L., & Sheng, J. (2019). The 12-item big three perfectionism scale for adolescents. Personality and Individual Differences, 151, 109, 536. doi:10.1016/j.paid.2019.109536

## Value of the Data

• Data collected from a paper-and-pencil questionnaire survey can be used for exploratory studies of the association between perfectionism and negative emotional symptoms, as well as wellbeing.
• The data was derived from a Chinese adolescent sample. It can be used for cross-cultural analysis and age comparisons.

## 1 Data description

The dataset includes Chinese adolescents’ responses to the following outcomes: (1) perfectionism, (2) flourishing and (3) negative emotion symptoms (i.e. depression, anxiety and stress). Additionally, the dataset comprises gender and age of the respondents. The data include responses from all 468 Chinese adolescents. Summary statistics of the sample was shown in Table 1.

**Table 1:** Descriptive statistics on a sample of Chinese adolescents’ responses to the items comprising the Big Three Perfectionism, Flourishing and Depression Anxiety Stress Scale (*n* = 468).

	Mean	Standard deviation	Median	Range	Skewness	Kurtosis	Shapiro-Wilk (p)	Cronbach's *α*
Age	14.49	1.11	14	13–17	0.31	−0.80	< 0.001	N/A
Rigid perfectionism	30.19	5.32	30	16–49	0.26	0.30	0.01	0.83
Self-critical perfectionism	49.46	9.29	49	23–82	0.07	0.29	0.40	0.88
Narcissistic perfectionism	35.58	8.14	36	17–64	0.36	0.23	< 0.001	0.86
Flourishing	40.22	6.23	41	15–56	−0.40	0.37	< 0.001	0.85
Negative Emotion Symptoms	15.3	8.94	14	1–59	0.99	1.57	< 0.001	0.88

The descriptive statistics of variables of different groups were shown in Table 2. The mean score of rigid perfectionism was 29.89 (SD = 4.99) in males and 31.28 (SD = 6.27) in females. The mean score of flourishing was 39.75 (SD = 6.27) in males and 41.85 (SD = 5.87) in females.

**Table 2:** Descriptive statistics and difference analysis of variables for different sub-groups.

		Gender		
	Total sample M (SD)	Male M (SD)	Female M (SD)	t
Age	14.49 (1.11)	14.49 (1.10)	14.50 (1.16)	−1.1
Rigid perfectionism	30.19 (3.52)	29.89 (4.99)	31.28 (6.27)	−2.04*
Self-critical perfectionism	49.46 (9.29)	49.70 (9.09)	48.57 (10.02)	1.08
Narcissistic perfectionism	35.58 (8.14)	35.15 (7.71)	37.13 (9.43)	−1.93
Flourishing	40.22 (6.23)	39.75 (6.27)	41.85 (5.87)	−3.01**
Negative Emotion Symptoms	15.3 (8.94)	15.71 (9.01)	13.76 (8.51)	1.95

* *p* < .05, ** *p* < .01

## 2 Experimental design, materials, and methods

### 2.1 Survey procedure

The samples were collected via interviews from a middle school in Sichuan Province, China. The dataset was obtained with paper-and-pencil questionnaire from May 2017 to October 2017 (368 males; mean age 14.49, *SD* = 1.11; range 13–17). All of the respondents were informed that the dataset was only used for research. The ethical statement and consent form was informed to all of the participants. All of the 468 participants provided valid data. The SPSS software was sued to calculate the summary statistics. The necessary ethical approval was obtained from the Department of Sociology, Wuhan University, China.

### 2.2 Measures

The 45-item Big Three Perfectionism Scale (BTPS) is a self-report scale including 3 subscales (i.e. rigid perfectionism, self-critical perfectionism and narcissistic perfectionism) [1]. Rigid perfectionism referred to individuals’ stubborn tendency to be flawless and without mistakes, which is made up by two facets (i.e., self-oriented perfectionism and self-worth perfectionism); Self-critical perfectionism captured evaluative concerns [1,2] and it was operationalized by four facets (i.e., concern over mistakes, doubts about actions, self-criticism, socially prescribed perfectionism). Narcissistic perfectionism subsumed four facets (i.e., other-oriented perfectionism, hypercriticism, entitlement, grandiosity), referring to one's belief that he or she is more outstanding than others and one's harsh standards towards people around [1]. Items were ranged from 1 (strongly disagree) point to 5 (strongly agree) point. The Cronbach's alpha of rigid perfectionism was 0.83, the Cronbach's alpha of self-critical perfectionism was 0.88 and the Cronbach's alpha of narcissistic perfectionism was 0.86. Duan, He, Huang & Sheng [3] developed a 12-item Big Three Perfectionism Scale as the short version of BTPS.

The Chinese version of Flourishing scale was employed [4,5]. Flourishing scale is made up by 8 items, which were paired with 7-Likert scale. Participants were required to rate each item, ranging from 1 (strongly disagree) to 7 (strongly agree). This scale has a good psychometric characteristic in various areas including China [4], [5], [6], [7], [8]. The Cronbach's alpha of the current sample was 0.85. The Cronbach's alpha of the Flourishing scale ranged from 0.90 to 0.93 in other samples.

Negative emotion symptoms was evaluated by the Chinese version of 21-item Depression Anxiety Stress Scale (DASS) [9]. DASS is commonly used to measure the negative emotional state such as depression, anxiety and stress [10]. It consisted of three subscales (i.e., Anxiety, Depression and Stress) with totally 21 items [11]. Each item was paired with a 4-point severity scale, respondents were requested to rate on each one, ranging from 0 (did not apply to me at all) to 4 (apply to me very much). Items describe the negative emotion symptoms (e.g. “I was unable to become enthusiastic about anything”) with a four point Likert scale. The Cronbach's alpha of the dataset was 0.88. The Cronbach's alpha of the Chinese version of 21-item Depression Anxiety Stress Scale were 0.83,0.80 and 0.82 for Depression, Anxiety and Stress subscales. The Cronbach's alpha of the total DASS was 0.92 [9].

## Appendix Supplementary materials

Image, application 1
